# Setting the stage for a Global Programme to Eliminate Lymphatic Filariasis: the first 125 years (1875–2000)

**DOI:** 10.1093/inthealth/ihaa061

**Published:** 2020-12-22

**Authors:** Eric A Ottesen, John Horton

**Affiliations:** Neglected Tropical Diseases Support Center, Task Force for Global Health, Decatur, GA 30030, USA; Tropical Projects, The Paddock, Hitchin SG4 9EF, UK

**Keywords:** albendazole, DEC, Global Programme to Eliminate Lymphatic Filariasis, ivermectin, lymphatic filariasis, World Health Organization

## Abstract

The development of the World Health Organization's Global Programme to Eliminate Lymphatic Filariasis (GPELF) can be interpreted through many different lenses—e.g. one focusing on the health or economic plight of affected individuals and populations, another tracking the individuals and organizations responsible for building the programme or, as in this review, one identifying each of the critical requirements and specific hurdles that need to be addressed in order to successfully construct the programme. For almost 75 y after the life cycle of LF was first described, the principal tool for countering it was vector control. Discovery that diethylcarbamazine (and later ivermectin and albendazole) could effectively treat affected and at-risk populations, along with the availability of a simple, field-based diagnostic test to monitor programme progress, provided the essential tools for LF elimination. Recognition of this potential by the global health community (including the World Health Assembly) led two pharmaceutical companies (GlaxoSmithKline and MSD (Merck & Co. Inc., Kenilworth, NJ, USA) to make enormous, unprecedented donations of albendazole and ivermectin to achieve this goal. Additional resource support from the public and private sectors and from health ministries in the 80 LF-endemic countries led to the creation of a Global Alliance to Eliminate LF, which launched the GPELF in 2000, just 125 y after the LF life cycle was first described.

## Defining the challenge

Creating a global programme is akin to mastering a complex puzzle, requiring a ‘big picture’ vision of the programme's potential goal and impact, tools capable of achieving that goal, strategies for using those tools, the energy (people, partnerships and resources) to propel the effort and a strategy for managing the whole (governance/leadership). But even more challenging than most puzzles, not all the puzzle pieces are available at the start, some must be created, and the shape of each missing piece is not fixed, but depends instead on the shape of the pieces created around it. Indeed, even the big picture vision of the project can change over time as new tools, strategies and opportunities become available. Such has been the fluidity in developing the GPELF, officially initiated in 2000 but building on a century of work before its lofty elimination goals of today could even be envisioned.^1^

## Assembling the tools

For the first 75 y following discovery of the lymphatic filariasis (LF) life cycle in the 1870s,^2^ programme targets were far less grandiose than those of today's GPELF. Although the filarial life cycle has always offered two potential targets for its interruption (infected people harbouring adult and microfilarial stages of the parasite and vector mosquitoes carrying larval and infective parasite stages), there were really no acceptable drugs for treating infected people. Since entomologic tools were available for mosquito control, reducing LF infection in the population through vector control became the principal goal and strategy of LF programmes—with, in many cases, very appreciable success.^3,4^

### Chemotherapy: diethylcarbamazine (DEC)

Everything changed, however, with the discovery in 1947 of DEC (Hetrazan, a derivative of the anthelminthic piperazine) for treating LF in humans.^5^ While its mechanism of action still remains uncertain, DEC is dramatically effective in clearing and killing microfilariae (mf) of both *Wuchereria bancrofti* and *Brugia malayi* and is largely effective in killing the adult worm stage as well. Its discovery ushered in an era in which chemotherapy became the predominant strategy for LF control, with one of its very earliest investigators being so impressed by its effectiveness that he even speculated in 1950 that if DEC were administered to entire at-risk populations, the goal of LF programmes might be able to shift from control to eradication!^6^

For decades thereafter, many of the approximately 80 LF-endemic countries used DEC as their principal tool for LF control, trying a remarkable variety of different treatment regimens and strategies—including single-day megadoses, weekly or monthly single doses, daily doses for weeks or months and even daily use of DEC as an additive in fortified table salt or other foods.^7^ While all of these approaches proved informative for developing a foundation for the GPELF, none was more valuable than the experiences from China.^8^

In the 1950s, China had clearly recognized the extent of the health and economic costs of LF on its national well-being and had prioritized the elimination of this disease. China's basic strategy was to test its at-risk populations for mf in the blood (particularly challenging because of the parasite's nocturnal periodicity), treat those individuals identified as mf-positive with ‘full-course’ DEC for multiple weeks and then ensure that the entire populations of its 15 endemic provinces (330 million people) either received single treatments of DEC or utilized DEC-fortified table salt routinely. These principal approaches were embedded in a highly organized and regimented framework of rigorous monitoring, evaluation, data management, logistics and social science.^8^ Importantly, China's programme also invested significantly in operational research, so that it could define both the required duration/dosages for DEC treatment and the critical epidemiologic thresholds for starting and stopping their programmes.

This information from the Chinese experience, along with observations from many other DEC-based LF programmes, proved essential for creating the GPELF. Japan, Korea, the Philippines, the Pacific Island countries, Malaysia, Thailand, Indonesia, India, Egypt and Brazil all initiated LF control programmes based on DEC administration.^7^ DEC was seen to be effective everywhere, but the optimal dose regimen for reducing mf or killing adult worms remained controversial until studies in the Pacific, Brazil and elsewhere showed that a single dose of DEC (6 mg/kg) was essentially equivalent to the same dose repeated daily for 2 or 3 weeks, with both regimens leading consistently to partial clearance of microfilaremia and partial killing of adult worms.^7,9,10^

### Ivermectin and albendazole

The next breakthrough that made the GPELF possible was the welcome development in the 1980s of two other drugs, ivermectin and albendazole, that could be administered safely and effectively in single doses either alone or with DEC to enhance DEC's partial effectiveness in clearing microfilaremia and killing adult parasites. In studies sponsored principally by the WHO's Special Programme in Tropical Disease Research (TDR) and MSD, single-dose ivermectin, which had been introduced as a safe microfilaricide for treating patients (and populations) with onchocerciasis,^11^ also proved to be a potent microfilaricide for LF at similar dosages.^12^ Then, fortuitously, albendazole, which had been developed by SmithKline Beecham (now GlaxoSmithKline) as a broadly effective anthelminthic,^13^ was found to rapidly kill adult worms of *W. bancrofti* after multiple doses.^14^ Although that initial multidose albendazole regimen induced unacceptable local inflammatory reactions around the dying worms,^14^ even single-dose albendazole was found in later TDR-supported studies to be effective in damaging the adult worms and inhibiting their production of mf,^15^ thereby decreasing microfilaremia in infected individuals through a different, and additive, mechanism from the direct killing of microfilariae by either DEC or ivermectin.

### Two-drug regimens

Thus, by the mid-1990s, three drugs, each with single-dose effectiveness against both *W. bancrofti* and *B. malayi* filariasis, had been found to be effective either alone or, for enhanced effectiveness, in two-drug combinations.^15,16^ The regimen of DEC + albendazole could be employed in most of the world where LF is endemic, but not in Africa. There onchocerciasis is frequently co-endemic with LF, and DEC often triggers intense inflammatory reactions around onchocerca microfilariae in the skin and, more importantly, the eyes; in these co-endemic countries it is ivermectin + albendazole that is safe for effectively treating LF. Formal safety studies of these two-drug combinations (albendazole + DEC or albendazole + ivermectin) were rapidly initiated by TDR and the WHO's Department for Control of Tropical Diseases (CTD). The studies proved that these two-drug regimens were equally safe as the single-dose regimens^17^ and therefore were ready to be employed as the preferred treatments for any global programme to eliminate LF. It would take almost two more decades before the value and safety of triple-drug regimens using these same medicines would be successfully explored.^18^

### Diagnostics

There are only two absolute requirements for defining a disease as eliminable: there must be a tool (treatment) effective enough to get rid of the disease and there must be a tool (diagnostic) effective enough to detect its presence (or its absence).^19^

Although microscopic detection of microfilaremia and serologic testing for antibodies were early standard diagnostics for LF, their difficulty for detection (e.g. nocturnal periodicity of blood microfilariae) and the lack of antibody sensitivity and specificity rendered them less than ideal for an elimination programme. Indeed, only after an effective antigen assay was developed (first in a laboratory and then in a point-of-care rapid-test format) could an LF elimination programme be advanced. Numerous antigen detection diagnostics were developed in the 1980s, but the ICT card test (ICT Diagnostics, Balgowlah, NSW, Australia), based on a monoclonal antibody to parasite antigen AD-12, became the standard for detecting *W. bancrofti* infections in the field.^20^ The ICT test transformed LF control programmes and made elimination of LF a feasible global target. Since the 20% of filarial infections caused by *B. malayi* are not detected by the ICT test, a ‘workaround assay’ based on detecting a unique (immunoglobulin G4) antibody was developed as a rapid diagnostic (Brugia Rapid). Because the presence of this antibody in the blood decreases rapidly when the infection is effectively treated, it can indirectly serve a similar role for programmes targeting *B. malayi*^21^ as the ICT antigen detection serves for bancroftian filariasis.

### Alleviating the suffering: morbidity management

It is not filarial infection but filarial disease—principally lymphedema, elephantiasis, hydrocele and acute adenolymphangitis—that makes LF so debilitating and stigmatizing (and recognizable). By the late 1990s, understanding the pathogenesis of LF disease had dramatically advanced. Ultrasound^22^ and lymphoscintigraphic^23^ techniques allowed, for the first time, visualization of both living adult worms and the localized lymphatic damage that they induce—lymphatic dilatation, compromised function and creation of foci for recurrent bacterial superinfection. Indeed, most important was the appreciation of how critical was the role of bacterial infection in the progression of filarial disease. This understanding truly changed the therapeutic outlook for affected patients from one of resignation and hopelessness to one where a regimen of intensive local hygiene, attentive limb care and prevention of bacterial and fungal infection can lead to dramatic patient improvement—many fewer acute inflammatory episodes, decreased limb size, freedom from the odour of chronic infections, a reduced sense of stigma and a greater sense of well-being.^24^ At the same time, surgical approaches for hydrocoele repair were also improved and then disseminated broadly throughout LF-endemic countries.^25^

## Developing strategies that utilize the tools available

The overall goal to eliminate LF was conceived as having two principal targets:^26^ to stop the spread of infection (by interrupting transmission) and to alleviate suffering caused by the disease (through morbidity management). Each required not only a how-to technical strategy, but also a non-technical, people-oriented strategy to ensure that the programme would be effectively implemented.

The technical strategy for interrupting transmission was agreed upon in the 1990s largely by WHO Expert Advisory Groups, academics, public health researchers and public- and private-sector health leaders through meetings hosted by the WHO's TDR and CTD departments.^26^ It was grounded in the practical experience from many countries with earlier, successful filariasis control programmes, especially those in China, Japan, Brazil, India and the Pacific Islands. While vector control (e.g. bed nets or environmental control) was recognized to be of value to LF elimination programmes and encouraged where feasible, the principal focus for interrupting transmission was to be on clearing infection from the human population. The approach targeted LF-endemic populations, first identifying them and then treating the entire eligible population with one of the two-drug regimens through annual mass drug administration (MDA) programmes for 4–6 y (the expected reproductive life span of the LF parasites).^26^ The many technical details—focusing on disease ‘mapping’; delineation of programme implementation areas; acquisition, management and distribution of medicines; population compliance; programme monitoring, evaluation and decision making—were captured in numerous guidelines and training materials organized by the WHO's CTD and TDR departments in advance of GPELF development.^27–32^

Similarly, for morbidity management, clinical and academic experts along with public- and private-sector health partners were convened by the WHO's TDR and CTD departments to develop and disseminate the programme's technical strategy. For lymphedema management, the principal strategy was to implement intensive hygiene and local care to prevent bacterial ‘superinfection’ of affected limbs through training of health workers, patients, their families and other caregivers. For managing hydrocele, the focus was on training of surgeons and providing education, access and rehabilitation for affected individuals. Again, practical guidelines and training materials were produced before the GPELF was launched.^33,34^

## Energizing the GPELF—people, partnerships and resources

Goals, tools and strategies are a global programme's *sine qua non*, but so too are people, partnerships and resources to propel the initiative. Technical tools and strategies will not function without people, but for people to be ready to engage they must first appreciate the value of the programme, believe that it can be successful, be assured that they will have partners to work with and, if they commit, be confident that they will have both the technical and financial resources necessary for success. For the GPELF, this confidence developed progressively in stepwise fashion during the 1990s.

### Step 1. 1993: International Task Force for Disease Eradication (ITFDE)

If there is a single stimulus that can be identified as the initiator of activities that most directly led to the GPELF, it is the 1993 determination by the ITFDE that LF was one of only six diseases it considered potentially eradicable with available tools.^35^ Constituted in 1988, the ITFDE's infectious disease and global health experts spent 4 y reviewing 94 infectious diseases for their potential eradicability. Assessed by the ITFDE in 1992,^36^ LF and five other diseases that had been reviewed earlier (guinea worm, polio, mumps, rubella and cysticercosis) were identified as the infectious diseases likely most amenable to eradication. This designation transformed the degree of international attention paid to LF. It also led to a new-found sense of urgency for the LF scientific community to hone its tools and strategies in preparation for possibly creating LF elimination programmes and to a new-found relevance within the global health community for LF prioritization, integration and impact assessment.

### Step 2. 1994–1997

While ITFDE recognition did not guarantee the future creation of programmes targeting LF elimination, the LF community immediately began to take the key steps necessary to address many of the unresolved elements (both technical and policy) that would be essential for effective programmes. These steps were taken at a series of meetings and research initiatives, highlighted below:

Principally technical

- 1994: ‘Informal consultation on new strategies for control of Lymphatic Filariasis’, WHO/CTD/TDR, Penang, Malaysia: 39 participants from 18 countries. A thorough review of ongoing national and local LF control programmes and the research studies associated with them, including the use of single-dose treatment with DEC and/or ivermectin; a consensus publication (‘Lymphatic filariasis infection and disease: Control strategies’)^37^ broadly endorsed the feasibility of LF elimination programmes.
- 1997: ‘The ICT Filariasis Test: a rapid-format antigen test for diagnosis of Bancroftian filariasis’.^20^ This seminal publication introduced the diagnostic rapid test that transformed thinking in WHO expert meetings and in implementation of most LF programmes.
- 1994–1997: Research studies and consensus meetings defining the effectiveness^38,39^ and safety^17^ of albendazole used with or without ivermectin or DEC as annual single-dose treatments in MDAs and optimizing the dosage and treatment regimens of ivermectin.^16^
- 1994–1997: Recognition of the dual determinants of lymphatic disease progression (lymphatic pathology plus bacterial superinfection) and its successful treatment^24,40^

Policy and implementation

- 1994–1997: A series of individual in-country consultations with ministries of health and WHO Regional Offices (Eastern Mediterranean [EMRO; Egypt ], Africa [AFRO; Chad, Ghana, Nigeria, Republic of Congo, Tanzania, Uganda], South-East Asia [SEARO; Bangladesh, India, Nepal, Sri Lanka] and Western Pacific [WPRO; China, Fiji, Malaysia, Philippines, Polynesia, Samoa, Thailand]) to explain and discuss potential national filariasis elimination programmes and a broader initiative targeting regional and global filariasis elimination.

By 1997 a general agreement had been reached on what a global LF elimination programme could look like both technically and from the perspective of national ministries of health.^26^

### Step 3. 1997: The World Health Assembly Resolution

While the ITFDE's designation of LF as an eradicable disease was the practical initiator of programmatic activities towards LF elimination, the real legitimizer of the GPELF was the 1997 World Health Assembly Resolution (WHA 50.29)^41^ that called on ‘Member States, agencies and organizations of the United Nations system, bi-lateral development agencies, non-governmental organizations and other concerned groups to mobilize support for global and national efforts to eliminate lymphatic filariasis as a public health problem’. This resolution had the immediate effect of putting LF on the health agendas of all endemic countries and turned the challenge for these countries from whether to embrace LF elimination as a priority target to one of how to embrace this elimination target. Furthermore, identifying ‘elimination’ as the target of this initiative set a bold goal for the programme that inspired donors and others to lend their support.

### Step 4. 1997–2000

After the WHA resolution in May 1997, the pace of creating the GPELF increased. Partnerships, resources, governance and technical guidance were all necessary ‘immediately,’ but perhaps the greatest urgency was for resources. Without at least start-up funding and access to the medicines that formed the basis of the programme's MDA strategy, the GPELF could not go forward.

### Resources

While SmithKline Beecham was enormously helpful early on in supporting many of the necessary administrative and technical costs involved in creating a global LF initiative, the first significant funding for programme implementation start-up came in October 1997 when the Arab Fund for Economic and Social Development pledged $5.4 million over 5 y to help establish LF elimination programmes in Arab Fund member countries in the WHO's EMRO. Since Egypt had had sophisticated LF control programmes for decades and had also played a key role in the research underpinning the GPELF's elimination strategy, its Ministry of Health along with the EMRO became important partners in creating the guidance and empirical data to document the effectiveness of the early programme implementation tools. This led others to provide additional critical resources—not only funding assurances (£3 million over 3 y from the UK Department for International Development), but even more essentially, medicines that were essential for the MDA strategy from pharmaceutical companies.

SmithKline Beecham had been involved in LF research since the 1980s when it showed the antifilarial effects of its anthelminthic drug albendazole.^14^ When albendazole's effectiveness in combination with either DEC or ivermectin was later recognized,^38^ SmithKline Beecham became enthusiastic about establishing a donation programme with the WHO to provide as much albendazole as needed by endemic countries to eliminate LF globally. Their commitment, signed with the WHO in December 1997, was an enormous expression of corporate philanthropy (totalling >9 billion donated tablets by 2020^42^), and it was all the more appreciated by national programmes because albendazole's broad anthelminthic activity already made it the preferred drug for school deworming programmes. Additionally, this donation, along with SmithKline Beecham’s other programme support, imparted a strength to the GPELF that truly assured its long-term growth and success.

Maximal effectiveness of the once-yearly MDA strategy to eliminate LF requires simultaneous administration of at least two antifilarial drugs.^15^ While DEC is very inexpensive, that is not the case for ivermectin (Mectizan). In 1987, MSD created a wholly new model and benchmark for corporate philanthropy when it agreed to donate Mectizan (through a non-governmental organization^43^ coordinating with the WHO) for as long as needed to treat onchocerciasis in all endemic countries. After the effectiveness of the albendazole and ivermectin two-drug regimen in LF programmes was realized,^38^ MSD agreed in 1998 to extend its already generous donation of Mectizan to include LF elimination programmes in all countries of Africa (plus Yemen) where LF is co-endemic with onchocerciasis.^44^

SmithKline Beecham (now GlaxoSmithKline [GSK]) and MSD established and have maintained a strong partnership with each other, the WHO, national LF and onchocerciasis programmes and both bilateral and non-government organizations helping to implement these MDA programmes. Indeed, it has been this strength of partnership that not only ensured a successful start with long-term success for the GPELF but also stimulated additional pharmaceutical donations of both DEC for LF programmes and other medicines for other diseases of underserved populations.^44^

### Governance and management

One of the most important and most challenging aspects of creating any large programme is to identify a governance and management structure that can focus on an agreed-upon goal and that can channel the enthusiasm and talent of all participants toward that goal without stifling individual and organizational ambition and creativity.

Immediately following the WHA resolution, the WHO took two approaches to establish a globally agreed-upon goal and governance structure. The first involved holding a technical Policy Retreat^45^ only weeks after the 1997 WHA resolution so that the WHO could affirm the overall strategic thinking that had led to this resolution and establish a Programme Review Group (PRG) of LF experts and national programme leaders to create the needed technical and administrative programmatic guidance. The second approach was to turn ‘outward’ towards current and potential stakeholders to seek their suggestions and their commitments to support this nascent global programme to eliminate LF. In October 1998, a large Partners Forum was held in Geneva, comprising almost 100 persons (including individuals with LF) representing a very broad range of endemic countries, international agencies, governmental and non-governmental organizations and different disciplines in global health.^46^ Over 3 d, the forum participants debated the value of LF elimination and hammered out potential solutions for key technical concerns, policy strategies, advocacy and funding challenges and numerous other potential barriers. The output from the forum and from the many supporting efforts around it^47^ were captured as a detailed programme strategic plan (‘Building Partnerships for Lymphatic Filariasis‘) that was published in 1999 and identified both specific commitments by each stakeholder and specific programmatic timelines for an envisioned GPELF.^48^

It was a six-member PRG appointed by the WHO's Director-General, along with support from the WHO secretariat and SmithKline Beecham representation, that was initially responsible for ensuring development of the mechanisms to support national LF programmes. Both MDA and morbidity management activities had to be provided for, in line with WHO guidance, and programmatic requirements for receiving the donation of medicines and for monitoring both therapeutic and programmatic effectiveness had to be established. Since four of the six PRG members were responsible for the LF programmes in their own countries, they became an enormously helpful group of ‘experimenters’ who could immediately evaluate the field effectiveness of new administrative guidelines, then review and refine them as needed.

During this pre-launch period of the GPELF, in addition to its administrative focus, the PRG was also responsible for ensuring that key technical elements of the programme were clearly defined. Most important was the PRG's assessment and approval of the extensive safety studies and regulatory evaluations to confirm that the two-drug regimens for the MDAs were no less safe than the already-accepted single-drug regimens.^17^ Similarly, the programme's epidemiologic integrity, the availability of epidemiologic tools and the rationale for their use in the initial assessment, monitoring, stopping and certifying of LF elimination all needed to be assured.^31^

## The launch

Finally, by the end of 1999, most of the necessary boxes had been ticked:^49^

- A single goal (global LF elimination) with two specific targets (interrupting transmission; alleviating suffering) had been established
- Tools (diagnostics, medicines) and strategies (MDAs, monitoring and evaluation, preventive hygiene) were available
- Feasibility, demonstrated in earlier programmes, was conceptually affirmed by expert opinion
- Global prioritization was mandated by the World Health Assembly resolution
- Enormous donations of medicines (albendazole from GSK and ivermectin from Merck) and ancillary support were pledged for as long as required for success
- Start-up funding had been secured from the Arab Fund and from the UK government
- A broad, supportive public–private partnership was established among endemic countries, bilateral and international agencies, foundations, non-governmental organizations, academia and private companies
- Technical and programmatic guidelines had been created and tested successfully for safety and effectiveness
- A governance structure for the programme had been agreed and established.

All that was left in 2000 was to ratify and officially launch both the WHO's GPELF and its supportive partnership, the Global Alliance to Eliminate LF (GAELF)—the former taking place in Manson House, London^50^ and the latter in Santiago de Compostela, Spain.^1^

## Data Availability

None.

## References

[1] Department of Communicable Diseases Control, Prevention and Eradication, World Health Organization. Eliminate Filariasis: Attack Poverty. Santiago de Compostela, Spain; 2000. Available from: https://www.who.int/lymphatic_filariasis/resources/who_cds_cpe_cee_2000.5/en/ [accessed 7 July 2020].

[2] Manson P. Further observations on Filaria sanguinis hominis. Med Rep Imperial Maritime Customs China. 1878. Special series No. 2 (14th issue):1–26.

[3] Sasa M. Human filariasis: a global survey of epidemiology and control. Baltimore: University Park Press; 1976.

[4] Webber RH. Eradication of *Wuchereria bancrofti* infection through vector control. Trans R Soc Trop Med Hyg. 1979;73(6):722–4. 395730 10.1016/0035-9203(79)90031-2

[5] Hewitt RI, White E, Wallace WS. Experimental chemotherapy of filariasis II. Effect of piperazine derivatives against naturally acquired filarial infections in cotton rats and dogs. J Lab Clin Med. 1947;32(11):1304–32. 18917890

[6] McGregor IA, Hawking F, Smith DA. The control of filariasis with Hetrazan; a field trial in a rural village (Keneba) in the Gambia. Br Med J. 1952;2(4790):908–11. 12978380 10.1136/bmj.2.4790.908 PMC2021811

[7] Ottesen EA. Efficacy of diethylcarbamazine in eradicating infection with lymphatic-dwelling filariae in man. Rev Infect Dis. 1985;7(3):341–56. 3895352 10.1093/clinids/7.3.341

[8] World Health Organization Regional Office for the Western Pacific, Control of Lymphatic Filariasis in China Editorial Board. Control of lymphatic filariasis in China. Manila: World Health Organization Regional Office for the Western Pacific; 2003. Available from: https://iris.wpro.who.int/handle/10665.1/1286 [accessed 9 July 2020].

[9] Sasa M. Pilot experiments in the control of bancroftian filariasis in Japan and Ryukyu. Bull World Health Org. 1963;28(4):437–54. 13986607 PMC2554734

[10] Noroes J, Dreyer G, Santos A et al. Assessment of the efficacy of diethylcarbamazine on adult *Wuchereria bancrofti in vivo*. Trans R Soc Trop Med Hyg. 1997;91(1):78–81. 9093637 10.1016/s0035-9203(97)90405-3

[11] Brown KR, Neu DC. Ivermectin – clinical trials and treatment schedules in onchocerciasis. Acta Leiden. 1990;59(1–2):169–75. 2198749

[12] Ottesen EA, Campbell WC. Ivermectin in human medicine. J Antimicrob Chemother. 1994;34(2):195–203. 7814280 10.1093/jac/34.2.195

[13] Horton J. Albendazole: a review of anthelmintic efficacy and safety in humans. Parasitology. 2000;121(Suppl):S113–32. 11386684 10.1017/s0031182000007290

[14] Jayakody RL, De Silva CSS, Weerasinghe WMT. Treatment of bancroftian filariasis with albendazole: evaluation of efficacy and adverse reactions. Trop Biomed. 1993;10:19–24.

[15] Gyapong JO, v Kumaraswami, Biswas G, Ottesen EA. Treatment strategies underpinning the Global Programme to Eliminate Lymphatic Filariasis. Expert Opin Pharmacother. 2005;6(2):179–200. 15757416 10.1517/14656566.6.2.179

[16] Brown KR, Ricci FM, Ottesen EA. Ivermectin: effectiveness in human filariasis. Parasitology. 2000;121(Suppl):S133–46. 11386685 10.1017/s0031182000006570

[17] Horton J, Witt C, Ottesen EA et al. An analysis of the safety of the single dose, two drug regimens used in programmes to eliminate lymphatic filariasis. Parasitology. 2000;121(Suppl):S147–60. 11386686 10.1017/s0031182000007423

[18] King CL, Suamani J, Sanuku N et al. A randomized trial of a new triple-drug treatment for lymphatic filariasis. N Engl J Med. 2018;379(19):1801–10. 30403937 10.1056/NEJMoa1706854 PMC6194477

[19] Ottesen E, Dowdle W, Fenner F et al. How is eradication to be defined and what are the biological criteria? In: Dowdle W,, Hopkins D, editors. The eradication of infectious diseases. Chichester, UK: Wiley; 1998, p. 46–59.

[20] Weil GJ, Lammie PJ, Weiss N. The ICT filariasis test: a rapid format antigen test for diagnosis of bancroftian filariasis. Parasitol Today. 1997;13(10):401–4. 15275155 10.1016/s0169-4758(97)01130-7

[21] Rahmah N, Taniawati S, Shenoy RK et al. Sensitivity and specificity of a rapid dipstick test (Brugia Rapid) in the detection of *Brugia malayi* infection. Trans R Soc Trop Med Hyg. 2001;95(6):601–4. 11816429 10.1016/s0035-9203(01)90091-4

[22] Amaral F, Dreyer G, Figueredo-Silva J et al. Live adult worms detected by ultrasonography in human bancroftian filariasis. Am J Trop Med Hyg. 1994;50(6):753–57. 8024070 10.4269/ajtmh.1994.50.753

[23] Witte MH, Jamal S, Williams WH et al. Lymphatic abnormalities in human filariasis as depicted by lymphoangioscintigraphy. Arch Intern Med. 1993;153(6):737–44. 8447712

[24] Dreyer G, Medeiros Z, Netto MJ et al. Acute attacks in the extremities of persons living in an area endemic for bancroftian filariasis: differentiation of two syndromes. Trans R Soc Trop Med Hyg. 1999;93(4):413–7. 10674092 10.1016/s0035-9203(99)90140-2

[25] World Health Organization/Department of Communicable Diseases Prevention, Control and Eradication. Surgical approaches to the urogenital manifestations of lymphatic filariasis. WHO/CDS/CPE/CEE/2002.33. Available from: https://www.who.int/lymphatic_filariasis/resources/who_cds_cpe_cee_2002.33/en/ [accessed 9 July 2020].

[26] Ottesen EA, Duke BOL, Karam M, Behbehani K. Strategies and tools for the control/elimination of lymphatic filariasis. Bull World Health Org. 1997;75(6):491–503. 9509621 PMC2487030

[27] UNDP/World Bank/WHO Special Programme for Research and Training in Tropical Diseases, WHO/UNICEF/Joint Programme for Health Mapping, WHO Division of Control of Tropical Diseases. Research on rapid geographical assessment of bancroftian filariasis. TDR/TDF/COMDT/98.2. Available from: https://apps.who.int/iris/handle/10665/63960 [accessed 9 July 2020].

[28] WHO/Department of Communicable Diseases Control, Prevention and Eradication. Preparing and implementing a national plan to eliminate lymphatic filariasis in countries where onchocerciasis in not co-endemic. WHO/CDS/CPE/CEE/2000.16. Available from: https://www.who.int/lymphatic_filariasis/resources/who_cds_cpe_cee_2000.16/en/ [accessed 9 July 2020].

[29] World Health Organization. Application from the ministry of health (country) to support a national programme to eliminate lymphatic filariasis. WHO/CDS/FIL99.1. Available from: https://apps.who.int/iris/handle/10665/66150 [accessed 9 July 2020].

[30] Gyapong JO, Kyelem D, Kleinschmidt I et al. The use of spatial analysis in mapping the distribution of bancroftian filariasis in four West African countries. Ann Trop Med Parasitol. 2002;96(7):695–705. 12537631 10.1179/000349802125001735

[31] World Health Organization. Report of a WHO informal consultation on epidemiologic approaches to lymphatic filariasis elimination: initial assessment, monitoring, and certification. WHO/FIL/99.195. Available from: https://apps.who.int/iris/handle/10665/66440?locale-attribute=ar&locale=fr&mode=full [accessed 9 July 2020].

[32] World Health Organization Lymphatic Filariasis Elimination Programme. Guidelines for certifying lymphatic filariasis elimination (including discussion of critical issues and rationale). WHO/FIL/99.197. Available from: https://www.who.int/lymphatic_filariasis/resources/who_fil_99.197/en/ [accessed 9 July 2020].

[33] World Health Organization/Department of Communicable Diseases Prevention, Control and Eradication. Lymphoedema staff manual: treatment and prevention of problems associated with lymphatic filariasis. Part 1: Learner's guide. WHO/CDS/CPE/CEE/2001.26a. Available from: https://www.who.int/lymphatic_filariasis/resources/who_cds_cpe_cee_2001.26a/en/ [accessed 9 July 2020].

[34] World Health Organization/Department of Communicable Diseases Prevention, Control and Eradication. Lymphoedema staff manual: treatment and prevention of problems associated with lymphatic filariasis. Part 2: Tutor's guide. WHO/CDS/CPE/CEE/2001.26b. Available from: https://www.who.int/lymphatic_filariasis/resources/who_cds_cpe_cee_2001.26b/en/ [accessed 9 July 2020].

[35] Centers for Disease Control and Prevention. Recommendations of the International Task Force for Disease Eradication. MMWR. 1993;42(RR-16):1–38. 8145708

[36] Ottesen EA. Potential eradicability of lymphatic filariasis. Wkly Epidemiol Rec. 1992;67:344–5.1419573

[37] World Health Organization Division of Control of Tropical Diseases, UNDP/World Bank/WHO Special Programme for Research and Training in Tropical Diseases. Lymphatic filariasis infection and disease: control strategies. TDR/CTD/FIL/PENANG/94.1. Available from: https://www.who.int/tdr/publications/tdr-research-publications/lymphatic-filariasis-infection/en/ [accessed 9 July 2020].

[38] World Health Organization Lymphatic Filariasis Elimination Programme. Report from informal consultation on albendazole research findings in lymphatic filariasis, 13–14 October 1998. WHO/FIL/98.194. Available from: https://www.who.int/lymphatic_filariasis/resources/who_fil_98.194/en/ [accessed 9 July 2020].

[39] Ottesen EA, Ismail MM, Horton J. The role of albendazole in programmes to eliminate lymphatic filariasis. Parasitol Today. 1999;15(9):382–6. 10461168 10.1016/s0169-4758(99)01486-6

[40] Shenoy RK, Sandhya K, Suma TK, Kumaraswami V. A preliminary study of filariasis related acute adenolymphangitis with special reference to precipitating factors and treatment modalities. Southeast Asian J Trop Med Public Health. 1995;26:301–5. 8629065

[41] World Health Assembly. Resolutions and decisions. WHA50.29: Elimination of lymphatic filariasis as a public health problem. Available from: https://www.who.int/neglected_diseases/mediacentre/WHA_50.29_Eng.pdf?ua=1 [accessed 9 July 2020].

[42] GlaxoSmithKline. Neglected tropical diseases: our commitment. Available from: https://www.gsk.com/media/4904/gsk-5500-ntd-factsheet-amend-p1_mp_2018-06-13.pdf [accessed 9 July 2020].

[43] Dull B. Mectizan donation and the Mectizan Expert Committee. Acta Leiden. 1990;59(1–2):399–403. 2378222

[44] Bradley M, Taylor R, Jensen J et al. Past, present and future: medicine donation programmes supporting the global drive to end the burden of neglected tropical diseases. Trans R Soc Trop Med Hyg. (Special Edition 2020) [in press]. 10.1093/trstmh/traa167 PMC7842096 33452881

[45] Seim AR, Dreyer G, Addiss DG. Controlling morbidity and interrupting transmission: twin pillars of lymphatic filariasis elimination. Rev Soc Bras Med Trop. 1999;32(3):325–8. 10380574 10.1590/s0037-86821999000300022

[46] Christakis AN, Dye KM, Post D. Case study: Partners’ Forum for designing a framework for the Global Programme to Eliminate Lymphatic Filariasis 1999. In: Flanagan TR,, Lindell CH, editors. The coherence factor – linking emotion and cognition when individuals think as a group. Charlotte, NC: Information Age Publishing; 2019, p. 89–102.

[47] World Health Organization Lymphatic Filariasis Elimination Programme . Lymphatic filariasis elimination: report of a meeting of the principals for the further enhancement of the public/private partnership, Amsterdam, The Netherlands, 28 May 1999. WHO/FIL/99.196. Available from: https://apps.who.int/iris/handle/10665/66442 [accessed 9 July 2020].

[48] World Health Organization Lymphatic Filariasis Elimination Programme. Building partnerships for lymphatic filariasis – strategic plan. WHO/FIL/99.198. Available from: https://www.who.int/lymphatic_filariasis/resources/who_fil_99.198/en/ [accessed 9 July 2020].

[49] Ottesen EA. The global programme to eliminate lymphatic filariasis. Trop Med Int Health. 2000;5(9):591–4. 11044272 10.1046/j.1365-3156.2000.00620.x

[50] Molyneux DH, Neira M, Liese B, Heymann D. Elimination of lymphatic filariasis as a public health problem. Lymphatic filariasis: setting the scene for elimination. Trans R Soc Trop Med Hyg. 2000;94(6):589–91. 11198635 10.1016/s0035-9203(00)90198-6

